# Bacillus Calmette–Guérin (BCG) Vaccination and the Immune–Brain Axis: Implications for Neuroprotection and Neurodegenerative Disease

**DOI:** 10.3390/vaccines14050412

**Published:** 2026-05-02

**Authors:** Magdalena Druszczynska, Beata Sadowska, Jakub Kulesza, Ewelina Kulesza, Marek Fol

**Affiliations:** 1 Department of Immunology and Infectious Biology, Faculty of Biology and Environmental Protection, University of Lodz, Banacha 12/16, 90-237 Lodz, Poland; beata.sadowska@biol.uni.lodz.pl (B.S.); marek.fol@biol.uni.lodz.pl (M.F.); 2 Department of Internal Diseases and Clinical Pharmacology, Medical University of Lodz, 91-347 Lodz, Poland; jakub.kulesza@mp.pl; 3 Department of Rheumatology and Internal Diseases, Medical University of Lodz, 90-549 Lodz, Poland; ewelina.kulesza@mp.pl

**Keywords:** BCG vaccine, trained immunity, neuroinflammation, immunomodulation

## Abstract

The Bacillus Calmette–Guérin (BCG) vaccine, originally developed for tuberculosis (TB) prevention, has recently attracted attention due to its broader immunomodulatory properties. In addition to its role in TB control, BCG induces trained immunity, a process involving epigenetic and metabolic reprogramming of innate immune cells that leads to altered systemic inflammatory responses. Increasing evidence suggests that these long-term immune adaptations may influence the central nervous system by modulating microglial activation and neuroinflammatory pathways implicated in neurodegenerative diseases. In parallel, chronic infections such as TB are associated with persistent systemic inflammation and immune dysregulation, which may contribute to microglial priming and increased vulnerability to neurodegeneration. This narrative review, based on a targeted literature search of PubMed, Scopus, Web of Science, Embase, and relevant preprint servers, synthesizes current evidence on the relationships between BCG vaccination, trained immunity, and neuroimmune interactions. We focus on studies addressing systemic immune reprogramming, microglial responses, and neuroinflammatory mechanisms relevant to neurodegenerative disorders. The available data suggest that BCG-induced immune modulation may exert context-dependent effects on the brain, with potential neuroprotective implications under certain conditions. However, the evidence remains heterogeneous and largely observational, and causality cannot yet be established. Further mechanistic and prospective studies are required to clarify whether BCG-induced trained immunity can modify the risk or progression of age-related neurodegenerative diseases.

## 1. Introduction

The Bacillus Calmette–Guérin (BCG) vaccine, introduced in 1921 for tuberculosis (TB) prevention, remains the most widely administered vaccine worldwide and a cornerstone of TB control [1]. Although it effectively protects against severe childhood TB, its efficacy against adult pulmonary disease is variable, which has driven the development of next-generation TB vaccines [2]. Beyond pathogen-specific protection, BCG exerts broad immunomodulatory effects attributed to trained immunity—epigenetic and metabolic reprogramming of innate immune cells that enhances responses to secondary stimuli [3,4,5]. This phenomenon has been demonstrated in experimental and clinical studies, with sustained changes in cytokine production, chromatin accessibility, and cellular metabolism [4,6]. Mechanistically, pathways such as NOD2 (Nucleotide-binding oligomerization domain-containing protein 2) and Akt (Protein kinase B)/mTOR (mechanistic Target of Rapamycin) signaling contribute to this long-term functional adaptation [2,3]. Epidemiological data further indicate reduced all-cause mortality and enhanced resistance to heterologous infections following BCG vaccination, effects not fully explained by adaptive immunity alone [4,5].

Increasing evidence indicates that systemic immune modulation influences central nervous system (CNS) function. Microglia, the resident innate immune cells of the brain, regulate neural homeostasis and mediate responses to injury and disease, while peripheral immune signals shape their activation states. Chronic inflammation is a key contributor to neurodegenerative disorders, including Alzheimer’s disease, Parkinson’s disease, and multiple sclerosis [6,7,8]. Experimental studies suggest that BCG vaccination modulates neuroimmune interactions, including macrophage polarization, neurotrophic factor expression, and cognitive outcomes [9,10]. However, these effects are context-dependent, as systemic immune activation may also exacerbate neuroinflammation [11,12]. The impact of next-generation TB vaccines on trained immunity and CNS outcomes remains poorly characterized.

The immune–brain axis supports dynamic communication between peripheral immunity and the CNS. Although the blood–brain barrier (BBB) maintains CNS homeostasis, it is a regulated interface that can be disrupted by aging, systemic inflammation, and neurological disease, allowing peripheral mediators and immune cells to influence neuroinflammatory processes [13,14,15,16,17]. Communication occurs via neural, humoral, and lymphatic pathways, while a properly functioning immune system contributes to CNS protection and repair [14,15,16,18,19,20,21,22,23]. These interactions involve cytokines, chemokines, and other signaling molecules [19,20,21,24,25,26,27]. Pro-inflammatory cytokines such as IL (interleukin)-1β, IL-6, and TNF (tumor necrosis factor)-α are central mediators of neuroinflammation and are associated with cognitive and behavioral alterations [19,21,24,28]. Microglia respond to these signals through context-dependent activation states that may either promote neurodegeneration or support repair [19,20,21,24,29].

**Figure 1:**
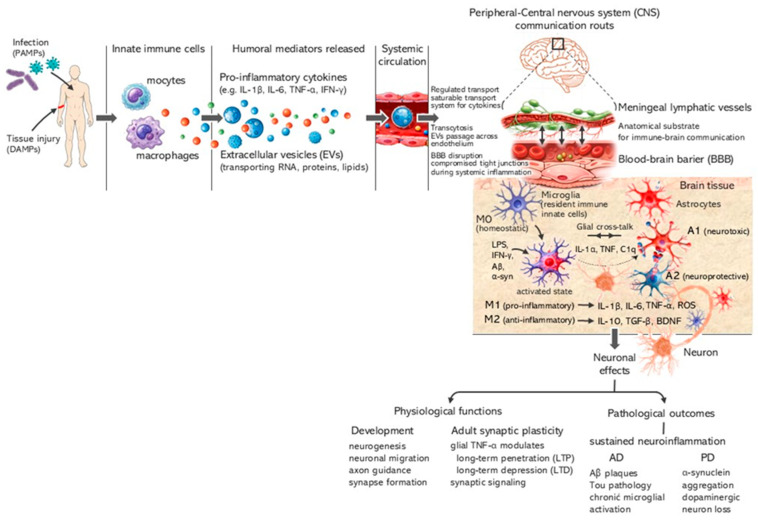
Immune–brain axis map. Peripheral inflammatory stimuli (for example, infection or tissue injury) activate monocytes and macrophages, leading to the release of pro-inflammatory cytokines and extracellular vesicles (EVs). These immune mediators communicate with the central nervous system (CNS) through several routes. Circulating cytokines can cross the blood–brain barrier (BBB) via regulated transport mechanisms or gain access when BBB integrity is compromised during systemic inflammation. EVs can also traverse the BBB by transcytosis. In addition, meningeal lymphatic vessels provide an anatomical substrate for immune–brain communication. Within the CNS, peripheral signals activate microglia, the resident innate immune cells, which in turn can induce reactive astrocyte phenotypes. Immune molecules are not only mediators of pathology but also key regulators of brain physiology. During development, cytokines influence neurogenesis, neuronal migration, axon guidance and synapse formation. In the adult brain, glia-derived cytokines such as TNF-α modulate activity-dependent synaptic plasticity, including long-term potentiation (LTP), long-term depression (LTD) and synaptic scaling, thereby shaping neural circuit refinement and cognition. Chronic dysregulation of these immune pathways contributes to sustained neuroinflammation and is implicated in neurodegenerative disorders such as Alzheimer’s and Parkinson’s disease (AD, PD). Illustration prepared based on information included in given references [22,23,30,31,32,33,34,35,36].

Taken as a whole, these findings support a bidirectional immune–brain axis and suggest that systemic immune reprogramming may influence neurodegenerative processes. In this context, BCG represents a clinically relevant model of long-term immune modulation. We hypothesize that BCG-induced trained immunity may alter microglial activation and neuroinflammatory pathways, thereby modifying susceptibility to age-related neurodegenerative diseases. This review integrates immunological, neurobiological, and epidemiological evidence to evaluate whether BCG and next-generation TB vaccines can influence CNS function through immune–brain interactions (Figure 1).

## 2. Materials and Methods

We conducted a narrative review based on a targeted literature search to identify studies addressing the immunomodulatory effects of BCG and next-generation TB vaccines, trained immunity, and their potential influence on the immune–brain axis. Electronic databases including PubMed, PubMed Central, Scopus, Web of Science, ScienceDirect, and Google Scholar were searched for relevant literature published up to January 2026. Only articles in English were considered. Conference abstracts without full text, letters to the editor, and non-peer-reviewed sources were generally excluded unless they provided essential conceptual insights. The search strategy combined terms related to tuberculosis vaccination, neuroimmunology, and neurodegeneration, including: “BCG”, “tuberculosis vaccine”, “next-generation TB vaccines”, “trained immunity”, “innate immune memory”, “microglia”, “astrocytes”, “neuroinflammation”, and “neurodegeneration”. Boolean operators (AND, OR) were used to refine the search, for example: (“BCG” OR “tuberculosis vaccine”) AND (“trained immunity” OR “innate immune memory”) AND (“microglia” OR “astrocytes”) AND (“neuroinflammation” OR “neurodegeneration”). Studies were selected based on their relevance to the scope of this review, with priority given to publications providing mechanistic, epidemiological, or clinical insights into: (i) BCG- or TB vaccine-induced systemic immune modulation, (ii) interactions between peripheral immunity and central nervous system cells, and (iii) links between chronic inflammation, TB, and neurodegeneration. Given the heterogeneity of the available evidence and the exploratory nature of this field, a formal systematic review framework was not applied. Instead, findings were synthesized qualitatively to provide an integrative overview of current knowledge and to highlight emerging hypotheses and research gaps. This narrative approach, while inherently susceptible to selection bias, was chosen to enable integration of mechanistic, epidemiological, and clinical evidence across a rapidly evolving and heterogeneous field.

## 3. Tuberculosis and Chronic Inflammation as a Biological Context for Neurodegeneration

TB represents a prototypical condition of sustained systemic inflammation, characterized by prolonged immune activation, chronic cytokine signaling, and persistent metabolic and epigenetic remodeling of innate and adaptive immune cells [37,38]. Even when pulmonary TB does not involve direct CNS infection, the systemic inflammatory milieu associated with chronic *Mycobacterium tuberculosis* (*M.tb*) exposure—including elevated TNF-α, IL-6, IFN (interferon)-γ, chemokines, and acute-phase proteins—can exert long-distance effects on the central nervous system [38,39]. Multiple studies have shown that chronic peripheral inflammation primes microglia into a sensitized, pro-inflammatory phenotype, lowering the threshold for exaggerated responses to subsequent insults such as infections, metabolic stress, β-amyloid accumulation, or α-synuclein pathology [31,40]. Such microglial priming is increasingly recognized as a fundamental accelerator of neurodegenerative cascades in Alzheimer’s and Parkinson’s diseases (AD, PD), promoting persistent neuroinflammation, oxidative stress, synaptic dysfunction, and impaired clearance of misfolded proteins [41,42].

TB therefore provides a biologically meaningful model for understanding how chronic inflammation interacts with neurodegenerative risk. Long-lasting immune perturbations associated with TB—including trained immunity–like epigenetic changes in monocytes and exhausted or dysregulated T-cell responses—could influence microglial behavior for years after infection resolution [43,44]. Epidemiological evidence suggests that individuals with a history of TB, especially untreated or recurrent disease, show elevated rates of cognitive impairment and neurological morbidity later in life, supporting the hypothesis that systemic infection leaves a prolonged neuroimmune imprint [45,46]. Furthermore, TB-induced metabolic inflammation overlaps with pathways implicated in neurodegeneration, including mTOR signaling, mitochondrial dysfunction, glycolytic rewiring of immune cells, and chronic NLRP3 (NLR family pyrin domain containing 3) inflammasome activation [31,47].

Understanding TB as a chronic inflammatory condition offers additional insight into why BCG vaccination—or new TB vaccines—might influence neurodegenerative trajectories. If TB itself contributes to long-term microglial priming and neuroimmune dysregulation, then preventing TB or altering host–mycobacteria interactions may reduce one upstream contributor to neurodegenerative susceptibility [44,48]. Conversely, BCG-induced trained immunity appears to recalibrate the very pathways dysregulated by chronic TB: it normalizes innate immune set points, reduces basal inflammation, enhances inflammation resolution, and modulates cytokine responses to secondary infections [43,49]. Thus, TB and BCG operate on a shared immunological landscape, but in opposite directions-TB amplifies chronic inflammation, while BCG appears to rebalance it. This framework strengthens the rationale for investigating BCG and next-generation TB vaccines as modulators of the immune–brain axis. It suggests that vaccines may not only protect against infection but also counteract the long-term immunological consequences of chronic *M.tb* exposure. Moreover, in high-incidence regions where TB and latent TB infection are common, understanding how persistent mycobacterial antigens shape neuroimmune biology may be key to accurately predicting the neuroprotective potential of BCG and its successors [50,51]. In this context, TB provides both the pathological counterpoint and the comparative model necessary to interpret how anti-TB vaccination—through trained immunity, adaptive rebalancing, and microglial modulation—might ultimately influence neurodegenerative disease risk across populations.

### Tuberculosis: Immunopathology and “Systemic Spillover” in the Context of Neuroinflammation

**Figure 2:**
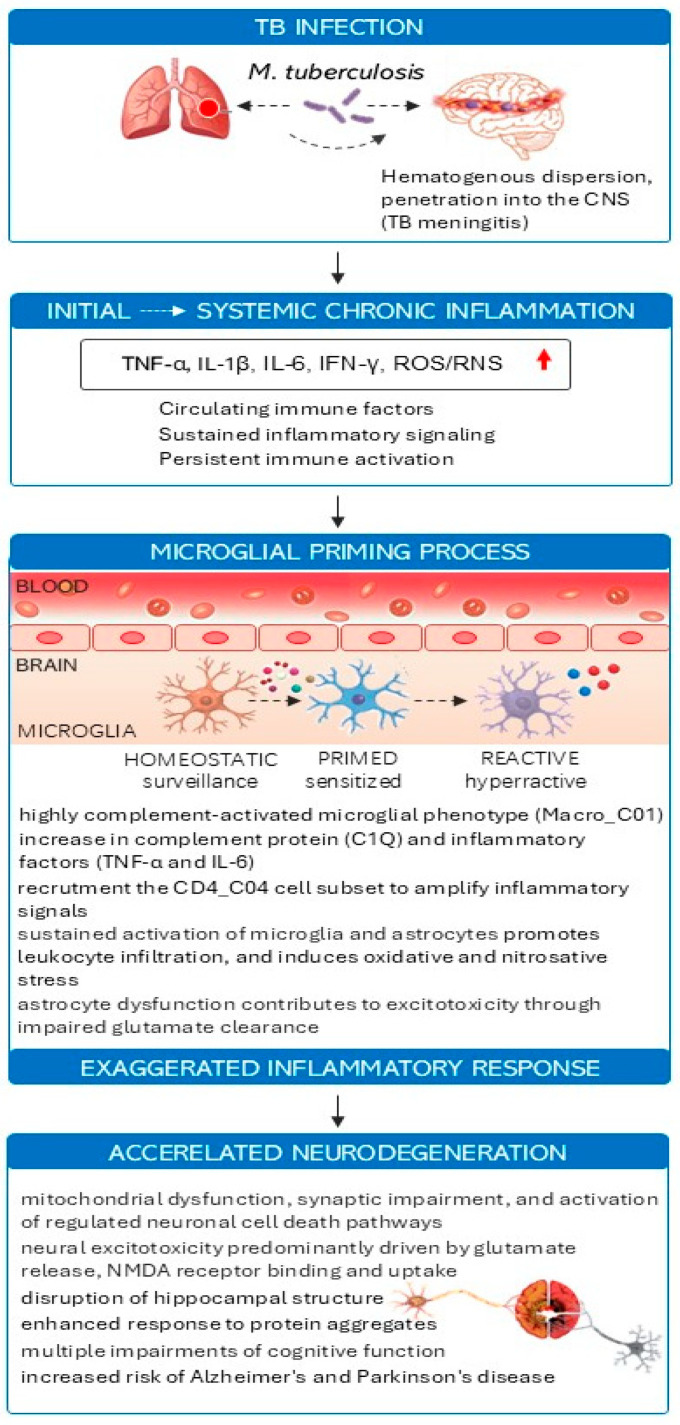
Potential impact of TB infection on the development of neurological changes in the central nervous system. TB infection initiates a cascade leading to accelerated neurodegeneration through microglial priming. TB-induced meningitis (TBM) most often arises from the reactivation of a previous pulmonary infection, although in some cases it may occur during a primary infection. Spreading through the bloodstream, *M.tb* can reach the brain or spinal cord, where they cause small inflammatory lesions located in subcortical areas or within the meninges. TBM causes blood–brain barrier (BBB) disruption, peripheral immune system infiltration, and the release of proinflammatory cytokines (TNF-α, IL-1β, IL-6, IFN-γ). Persistent inflammatory signals induce microglial activation. In pediatric TBM, single-cell transcriptomics reveal microglial phenotypes with a high degree of complement activation. Activated microglia exhibits an excessive inflammatory response. These events cause direct damage to neurons through cytokine neurotoxicity and oxidative stress, synaptic dysfunction, chronic BBB disruption, and exacerbation of pre-existing neurodegenerative pathology. The persistent neuroinflammatory environment accelerates neurodegeneration, manifested by cognitive impairment and an increased risk of long-term neurodegenerative diseases. Understanding this TB–neuroinflammation–neurodegeneration axis may help identify therapeutic targets to prevent neurological sequelae after infection. Illustration prepared based on information included in given references [45,57,58,59,60].

TB provides a model for studying the interplay between chronic infection, systemic inflammation, and neuroimmune modulation. The host immune response to TB progresses from acute to chronic phase, each with distinct immunopathological characteristics. In the acute phase, alveolar macrophages, dendritic cells, and neutrophils recognize *M.tb* via pattern recognition receptors (PRRs), including Toll-like receptor (TLR)2, TLR4, and NOD2, initiating strong pro-inflammatory signaling [37,38]. Cytokines such as TNF-α, IL-1β, IL-6, and IFN-γ recruit additional immune effectors, activate microbicidal pathways, and induce local tissue inflammation. While these responses are crucial for early containment of *M.tb*, excessive or uncontrolled activation contributes to tissue damage and systemic cytokine release (Figure 2).

When infection is not eradicated, the immune system forms granulomas, organized aggregates of macrophages, epithelioid cells, lymphocytes, and multinucleated giant cells, which function to sequester bacilli and prevent dissemination [52]. Granulomas, however, are not inert: they continuously produce cytokines, chemokines, matrix metalloproteinases (MMPs), and other inflammatory mediators that enter systemic circulation, a phenomenon described as “systemic spillover” [43,44]. Chronic low-grade inflammation resulting from this “spillover” can prime peripheral monocytes and hematopoietic progenitors, inducing epigenetic and metabolic remodeling analogous to trained immunity, yet potentially maladaptive if inflammation persists [48,53]. “Systemic spillover” has important implications for the CNS. Circulating cytokines can influence the BBB, increasing its permeability, and modulate microglial activation even without direct CNS infection [31,54]. Chronic exposure to TNF-α, IL-1β, and IFN-γ sensitizes microglia to subsequent insults, a process known as microglial priming, which lowers the activation threshold and amplifies neuroinflammatory responses to secondary stimuli such as metabolic stress, protein aggregates, or infections [40,42]. This establishes a mechanistic link between peripheral induced by latent TB and the potential acceleration of neurodegenerative processes, including Alzheimer’s and Parkinson’s diseases. Furthermore, TB-induced metabolic and epigenetic changes intersect with pathways implicated in neurodegeneration. Chronic inflammation affects mitochondrial function, induces oxidative stress, activates the NLR family pyrin domain containing (NLRP)3 inflammasome, and promotes maladaptive microglial responses [48]. In this context, granulomas act as both protective structures and persistent sources of pro-inflammatory signals, establishing a chronic immunological milieu with potential CNS consequences. Thus, latent or pulmonary TB without direct CNS infection, sustained systemic cytokine release can prime CNS innate immunity, supporting the concept that chronic peripheral inflammation contributes to long-term neuroimmune alterations.

In cases of tuberculous meningitis (TBM), *M.tb* directly invades the CNS, producing a local cytokine storm characterized by elevated TNF-α, IL-6, IFN-γ, chemokines, and MMPs in cerebrospinal fluid [55]. This localized neuroinflammation causes BBB disruption, astrocytic activation, oxidative stress, and neuronal injury. TBM serves as a natural model for studying CNS inflammatory responses to mycobacterial infection, demonstrating how pathogen-induced cytokine networks drive microglial activation and neurodegeneration-like pathology [56]. Studying TB and TBM in this framework emphasizes why BCG vaccination and next-generation TB vaccines may have neuroprotective potential. By preventing primary TB infection, reducing systemic inflammation, or inducing beneficial trained immunity, these vaccines could mitigate microglial priming and downstream neuroinflammatory cascades [44,49]. TB thus exemplifies how peripheral infection and chronic inflammation can shape CNS immunity, providing a critical experimental and conceptual model for understanding neuroimmune interactions relevant to neurodegenerative diseases.

## 4. Relationship Between Tuberculosis and Neurodegeneration (Epidemiology and Clinical Evidence)

Neurodegenerative diseases, including dementia, Alzheimer’s disease, Parkinson’s disease, amyotrophic lateral sclerosis, Huntington’s disease, and Friedreich’s ataxia, are progressive and currently incurable disorders characterized by selective neuronal loss. Despite their growing global burden, the factors driving their onset and progression remain incompletely understood [61]. Increasing evidence suggests that survivors of TB have a higher risk of developing neurodegenerative diseases, particularly dementia and Parkinson’s disease, indicating long-term effects of TB-induced immune responses on the immune–brain axis [46,62]. Among TB manifestations, tuberculous meningitis shows the strongest association with subsequent neurodegeneration [63]. Importantly, inflammatory activation may persist after microbiological cure, potentially contributing to chronic neuroimmune dysregulation [64,65].

While both *M.tb* infection and BCG exposure can activate overlapping innate immune pathways, their immunological context and systemic consequences may differ substantially (Table 1). Pulmonary TB represents a chronic infection characterized by sustained inflammation, prolonged antigenic stimulation, tissue damage, and potential neuroinflammatory sequelae driven by persistent cytokine dysregulation and possible blood–brain barrier disruption [66,67]. Such a chronic inflammatory milieu may promote microglial priming, sustained activation of innate immune pathways, and long-term neuroinflammatory signaling, all of which are increasingly recognized as contributors to neurodegenerative processes. In addition, prolonged immune activation during TB may lead to immune exhaustion and maladaptive inflammatory states, further exacerbating tissue damage and impairing immune resolution. In contrast, intravesical BCG is a controlled, localized immunotherapy administered in a compartmentalized setting, which, despite inducing systemic immune activation, does not involve chronic pathogen persistence [68]. Importantly, BCG-induced immune activation is typically transient and regulated, and may promote a form of balanced innate immune reprogramming rather than sustained inflammation. This controlled immune stimulation has been associated with systemic immune recalibration, which may contribute to protective effects extending beyond the primary site of administration. Differences in host immune status, disease burden, and treatment context further contribute to these divergent outcomes. Moreover, TB is frequently associated with socioeconomic and clinical confounders—such as comorbidities, nutritional status, and limited healthcare access—that may independently increase neurodegenerative risk. Conversely, patients receiving intravesical BCG are typically under close clinical surveillance and represent a more selected population. Although both exposures may induce elements of trained immunity, the magnitude, duration, and inflammatory tone of immune reprogramming are likely distinct: chronic TB may promote maladaptive or exhausted immune responses, whereas BCG induces a more controlled form of innate immune training with potentially beneficial immunomodulatory effects [46,62,65,69,70,71,72,73,74]. Taken together, these observations suggest that chronic infection–driven inflammation and immune dysregulation in TB contrast with the controlled, transient, and potentially immunoregulatory effects of BCG therapy, which may explain their opposing associations with neurodegenerative outcomes.

However, epidemiological evidence remains limited by methodological heterogeneity and long latency between infection and disease onset. Reliable biomarkers are therefore needed to capture early neuroimmune changes and distinguish causality from confounding [75,76]. Although biomarkers and neuroimaging aid diagnosis [77,78,79], their interpretation is limited by low specificity, invasiveness, and confounding factors such as comorbidities and blood–brain barrier integrity [80,81,82]. These limitations contribute to the scarcity of prospective studies incorporating validated CNS markers in TB populations.

Another major challenge is the lack of standardization of BCG exposure. Variations in strain, dose, timing, and revaccination influence immune responses and trained immunity induction [43,83,84,85]. Intravesical BCG further increases heterogeneity due to differences in administration protocols [65]. Comorbidities such as diabetes and human immunodeficiency virus (HIV) also modulate inflammatory responses and may affect neurodegeneration risk [31,86,87], complicating causal interpretation [46]. Despite these limitations, epidemiological studies report consistent associations. TB has been linked to increased dementia risk (1.21-fold), Alzheimer’s disease and vascular dementia, and Parkinson’s disease (1.38-fold), particularly shortly after diagnosis [46,62,69]. Similar short-term cognitive risks are observed after other severe infections, and central nervous system TB may mimic primary dementia syndromes [88,89].

Mechanistically, TB induces systemic inflammation involving cytokines such as TNF-α, IL-6, IFN-γ, and IL-1β, which are also implicated in neurodegeneration [90]. However, when neurodegenerative disease is already established, a direct causal role of TB-related inflammation in disease onset appears less likely. Population-based data confirm an increased Parkinson’s disease risk following TB, particularly within the first year [46]. In contrast, experimental and clinical data suggest that BCG vaccination may exert neuroprotective immunomodulatory effects. In animal models, BCG improves cognitive function, reduces neuroinflammation, and increases neurotrophic and anti-inflammatory factors without significantly affecting amyloid burden [10]. These effects are associated with peripheral immune reprogramming and recruitment of anti-inflammatory monocytes. BCG-induced trained immunity, involving epigenetic and metabolic reprogramming of innate immune cells, may underlie these effects. Additionally, BCG has shown potential as an adjuvant in anti-amyloid immunotherapy, improving outcomes and reducing adverse effects [91].

Clinically, intravesical BCG therapy for bladder cancer induces systemic immune responses, including IL-2–mediated expansion of regulatory T cells with anti-inflammatory and potentially neuroprotective effects [70]. Cohort studies consistently report reduced risk of Alzheimer’s disease and related dementias in BCG-treated patients [65,71,72,73,74,92], with effects particularly evident in older individuals and, in some studies, women. However, heterogeneity in study design, populations, and treatment protocols limits definitive conclusions.

Overall, while TB is associated with increased neurodegenerative risk and BCG exposure with potential protection, the evidence remains heterogeneous and susceptible to confounding. Variability in exposure, host factors, and study design complicates interpretation. Well-designed prospective studies incorporating standardized exposure definitions, validated CNS biomarkers, and long-term follow-up are needed to clarify causal mechanisms and therapeutic potential.

## 5. BCG and Novel Tuberculosis Vaccines: Mechanisms of Potential Modulation of the Immune–Brain Axis

### 5.1. BCG—Beyond Tuberculosis: Systemic and Neuroimmune Effects

Although developed in the 1920s, the BCG vaccine remains a key model for studying trained immunity [93,94]. This concept describes long-lasting functional reprogramming of innate immune cells, which were traditionally considered devoid of memory, leading to altered responsiveness after exposure to specific stimuli [95]. This finding has reshaped the classical view of immunological memory and the concept of immunization [5,53,96,97]. Evidence shows that BCG effects extend beyond specific protection against TB, enhancing immune responses to unrelated bacterial, viral, and parasitic pathogens, including *Plasmodium falciparum* [98,99,100,101]. These effects are mediated by functional reprogramming of monocytes, macrophages, and Natural Killer (NK) cells [3,102,103].

Importantly, while *M.tb*–derived vaccine formulations can activate overlapping innate immune pathways, current evidence does not support a uniform induction of trained immunity across all vaccine types. Differences in antigenic composition, viability, dose, and adjuvant properties likely result in distinct immunological outcomes, and extrapolation between vaccine platforms should therefore be made with caution. In particular, comparison with other bacterial vaccines provides further insight into the specificity of these effects. Vaccines against tularemia or brucellosis also contain complex antigenic repertoires and can induce robust innate and adaptive immune activation [104,105]. However, antigenic complexity alone does not appear sufficient to induce durable trained immunity. In contrast to BCG, which is a live attenuated mycobacterial vaccine capable of transient intracellular persistence and broad pattern recognition receptor engagement, the capacity of these vaccines to induce sustained epigenetic and metabolic reprogramming of innate immune cells remains less well established. These differences suggest that heterologous effects observed with BCG are more likely related to its biological properties and quality of innate immune stimulation than to antigenic complexity alone.

A major advancement in this field is the recognition that trained immunity is initiated not only in circulating monocytes but also at the level of hematopoietic stem and progenitor cells (HSC/HSPC) in the bone marrow [106,107]. Sun et al. demonstrated that BCG vaccination induces epigenetic remodeling of HSC/HSPC, including increased chromatin accessibility at loci regulating inflammatory responses (e.g., histone H3 lysine 4 trimethylation (H3K4me3), histone H3 lysine 27 acetylation (H3K27ac)), resulting in a persistent transcriptional program associated with trained immunity [106]. This is accompanied by expansion of myeloid lineages and enhanced production of IL-1β, TNF, and IL-6 upon secondary stimulation. These changes display features of long-term immune memory and align with earlier findings on monocyte epigenetic reprogramming after BCG exposure [2].

However, trained immunity is not exclusively defined by increased production of canonical pro-inflammatory cytokines such as TNF-α, IL-1β, IL-6, and IFN-γ. Although these cytokines are widely used as functional readouts, they represent only one component of a broader biological program that includes epigenetic remodeling, metabolic rewiring, and transcriptional reprogramming [108]. Moreover, cytokine elevation may also reflect non-specific innate immune activation, including adjuvant-driven effects, rather than stable long-term immune memory.

IL-1β appears to be a central mediator of this process, linking vaccination to myeloid progenitor expansion and differentiation [109]. Blockade of IL-1 signaling abolishes trained immunity in HSC/HSPC, confirming its key regulatory role, consistent with studies identifying IL-1 as a critical driver of innate immune memory [106,110,111]. In parallel, trained immunity is tightly linked to metabolic rewiring, including enhanced glycolysis via the mTOR– Hypoxia-Inducible Factor 1 (HIF-1) pathway and accumulation of metabolites such as fumarate and mevalonate, which support epigenetic modifications through chromatin-modifying enzymes [112,113]. Together, these processes establish a state of sustained “inflammatory readiness,” increasing the functional capacity of monocytes, macrophages, and NK cells. HSC/HSPC therefore act as central regulators of innate immune adaptation by integrating environmental signals, biasing myelopoiesis, and maintaining immunological imprinting over extended periods [114,115,116]. These advances in understanding trained immunity at the level of bone marrow progenitors and innate effector cells have opened new perspectives on long-term immune modulation within the central nervous system, particularly regarding the potential involvement of microglia and astrocytes.

### 5.2. Next-Generation TB Vaccines as Modulators of Systemic and Neuroimmune Responses

Next-generation TB vaccine candidates, including recombinant BCG strains, viral-vectored vaccines, subunit formulations, and nucleic acid–based platforms, are designed not only to improve protection against *M.tb* but also to induce broader immunological reprogramming. Increasing evidence indicates that these approaches may differentially modulate adaptive and innate immune responses, thereby influencing systemic inflammatory networks and potentially engaging pathways relevant to the immune–brain axis. Through shaping peripheral immunity, they may indirectly affect microglial priming, astrocytic function, and neuroinflammatory signaling, although the magnitude and durability of these effects vary substantially between vaccine classes.

Among subunit vaccine approaches, M72/AS01E is a recombinant antigen-based formulation composed of the M72 fusion protein (Mtb32A and Mtb39A) combined with the AS01E adjuvant system (MPL and QS-21). It induces strong T-helper (Th)1/Th17-skewed immune responses and promotes polyfunctional CD4^+^ T cells producing IFN-γ, IL-2, and TNF-α, which are considered correlates of protection against TB [117,118,119,120]. Similarly, H56:IC31 combines multiple antigens (Ag85B, ESAT-6, Rv2660c) with the IC31 adjuvant and induces durable Th1-type immunity characterized predominantly by IFN-γ, TNF-α, and IL-2 production [121]. While these vaccines primarily act through adaptive immunity, adjuvant-driven activation of innate immune receptors may transiently influence monocyte and dendritic cell function; however, evidence for stable, long-term trained immunity comparable to BCG remains limited [117,118,119,120,121]. Importantly, current studies on these subunit vaccines remain restricted to immunogenicity and safety endpoints, and no data exist linking their administration to neuroinflammatory changes, cognitive outcomes, or neurodegenerative disease risk.

**Figure 3:**
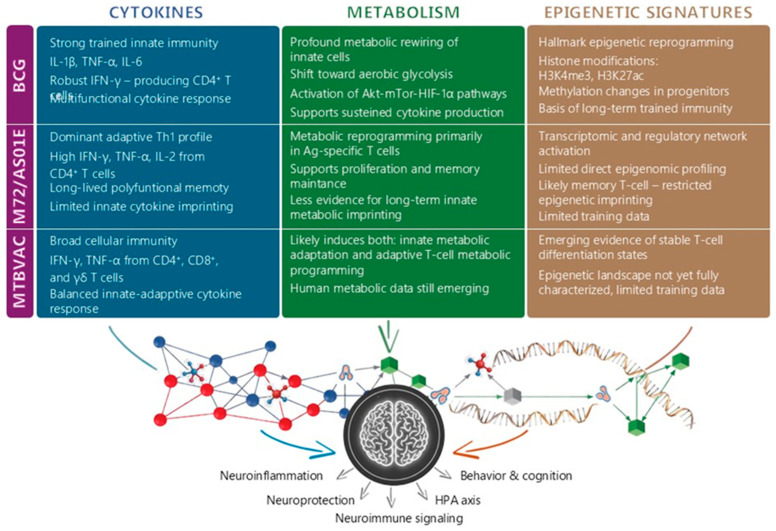
Comparative immune profiles of BCG, M72/AS01E, and MTBVAC vaccines. BCG induces robust trained immunity characterized by innate cytokine production, metabolic rewiring toward aerobic glycolysis, and epigenetic reprogramming. M72/AS01E elicits strong Th1-based adaptive immune responses with limited evidence of durable innate immune imprinting. MTBVAC generates broad cellular immunity combining adaptive and innate components, with emerging experimental evidence of metabolic and epigenetic reprogramming. Peripheral immune signals from cytokines, metabolic mediators, and epigenetically programmed immune cells can influence the CNS homeostasis through blood–brain barrier (BBB) signaling, neural pathways, and hypothalamic–pituitary–adrenal (HPA) axis activation. Figure summarizes immunological mechanisms based on available literature [3,118,125,133,143,144].

Live attenuated vaccine platforms such as VPM1002 and MTBVAC engage broader immunological mechanisms involving both adaptive and innate immunity (Figure 3) [122,123,124,125,126]. VPM1002, a recombinant BCG strain expressing listeriolysin O, enhances cytosolic antigen processing and promotes stronger CD4+ and CD8+ T-cell responses, alongside increased IL-1β and IL-18 production in preclinical studies [127,128]. MTBVAC, a live attenuated *M.tb* vaccine retaining the full antigen repertoire, induces robust Th1 responses and displays epigenetic and metabolic signatures consistent with trained immunity, including enhanced glycolysis, glutaminolysis, and histone modifications at promoters of inflammatory genes [118,129,130,131]. Early clinical trials confirm its safety and strong immunogenicity, with IFN-γ-producing CD4+ T-cell responses comparable or superior to BCG, supporting its potential as a next-generation platform capable of broader immune reprogramming [103,131]. However, despite these mechanistic features, there is currently no clinical or epidemiological evidence that MTBVAC or VPM1002 influence neurodegenerative disease incidence, progression, or CNS inflammatory biomarkers in humans or experimental models.

DNA and mRNA-based TB vaccine platforms have also emerged as promising candidates due to their high immunogenicity and ability to induce strong antigen-specific CD4+ and CD8+ T-cell responses [132]. These vaccines rely on in situ antigen expression and efficient immune presentation, resulting in potent but highly targeted adaptive immunity. However, their capacity to induce trained immunity remains uncertain. Current evidence does not support the induction of classical trained immunity, defined as long-term functional reprogramming of innate immune cells mediated by stable epigenetic and metabolic changes [133]. In contrast to live attenuated vaccines such as BCG, which consistently induce such innate immune memory in monocytes and NK cells, nucleic acid–based platforms have not been shown to generate durable innate immune reprogramming. Although transient innate immune activation may occur following vaccination—potentially mediated by pattern recognition receptor signaling triggered by plasmid DNA sensing pathways or lipid nanoparticle–associated mRNA delivery—these effects are consistent with acute innate immune stimulation rather than stable trained immunity. Therefore, DNA and mRNA TB vaccines should currently be considered primarily adaptive immunity–oriented platforms, with no confirmed evidence of long-term innate immune memory induction or CNS-related effects.

Overall, while BCG remains the prototypical inducer of trained immunity, evidence indicates that next-generation TB vaccines differ substantially in their ability to induce long-term innate immune reprogramming. These differences likely reflect variation in antigen complexity, vaccine viability (live versus non-live platforms), adjuvant composition, and intracellular persistence. As a result, their downstream effects on systemic inflammation and immune–brain communication are unlikely to be uniform. Importantly, cytokines such as IL-1β, TNF-α, and IL-6 remain central mediators linking peripheral immune activation to CNS processes, including blood–brain barrier integrity, microglial activation states, and neuroinflammatory signaling cascades [134,135,136]. Variability in cytokine induction across vaccine platforms may therefore have implications for immune–brain signaling; however, no evidence currently supports a direct link between next-generation TB vaccines and neurodegenerative disease modulation. In this context, it is critical to emphasize that all available data on M72/AS01E, MTBVAC, VPM1002, and related candidates are derived from immunological endpoints only (safety, reactogenicity, adaptive immune responses), and no studies have directly evaluated outcomes related to Alzheimer’s disease, Parkinson’s disease, or other neurodegenerative disorders. Therefore, any potential effects on CNS function remain speculative and mechanistically inferred rather than experimentally demonstrated.

Beyond classical immunological effects, BCG has been associated with systemic immunomodulatory and neuroimmune effects that extend beyond a purely pro-inflammatory paradigm. Clinical studies in healthy volunteers have shown that BCG vaccination can reduce basal systemic inflammatory markers despite enhancing cytokine responsiveness upon ex vivo stimulation, suggesting a regulatory form of immune reprogramming rather than sustained inflammation [137,138]. This immunomodulatory profile appears to be influenced by host factors, including sex-dependent differences in inflammatory regulation. In line with this, longitudinal observations indicate that BCG vaccination may reduce circulating pro-inflammatory cytokines, chemokines, and acute-phase proteins, supporting its role in dampening chronic low-grade inflammation rather than amplifying it [139]. Such systemic immune recalibration is particularly relevant in the context of aging-associated inflammatory states, which are strongly linked to neurodegenerative susceptibility.

Importantly, experimental models further suggest that BCG-induced immune modulation may extend to the central nervous system. Combined BCG vaccination and environmental enrichment enhances hippocampal neurogenesis and cognitive performance, accompanied by increased levels of neurotrophic factors such as BDNF Brain-Derived Neurotrophic Factor) and IGF-1 (Insulin-like Growth Factor 1) and a shift in microglial polarization toward an anti-inflammatory M2 phenotype [140]. Similarly, neonatal BCG exposure has been shown to confer long-term resistance to inflammation-induced behavioral and cognitive deficits, associated with reduced pro-inflammatory cytokines and increased neurotrophic signaling across brain regions [141]. These effects are supported by evidence of enhanced hippocampal neurogenesis, increased expression of M2-associated markers (Arginase-1 (ARG1), Chitinase-like protein 3 (Ym1)), and improved behavioral outcomes, indicating that early-life immune stimulation may induce durable immune–brain axis reprogramming with functional consequences for neuroplasticity and cognitive resilience [142].

### 5.3. Microglia and Astrocytes as Mediators of Vaccine-Driven CNS Modulation

Microglia and astrocytes are key cellular sensors of peripheral immune signals and represent central mediators linking systemic immunomodulation to CNS outcomes. Peripheral immune activation, including vaccination-induced trained immunity, can alter glial activation states, cytokine production, and neuroinflammatory signaling. These CNS-resident cells therefore act as indirect targets of BCG and next-generation TB vaccines, translating systemic immune cues into functional changes within the brain. Such modulation may influence synaptic function, neuronal survival, and susceptibility to neurodegenerative disease, highlighting a key immune–brain axis pathway.

Microglia serve as principal intermediaries of peripheral immune signaling in the CNS, while astrocytes actively participate in immune signal propagation and regulation rather than serving solely supportive functions [145,146,147]. Both cell types dynamically respond to endogenous pathological proteins (amyloid-β (Aβ), α-synuclein, Tau) and exogenous stimuli (lipopolysaccharide (LPS), cytokines), adjusting cytokine secretion and shaping the neuronal microenvironment [148,149]. Microglia constitute approximately 10–15% of brain cells and function as resident macrophages responsible for immune surveillance, synaptic remodeling, and clearance of cellular debris [150,151].

Importantly, microglial development follows a tightly regulated temporal program, which defines critical windows of heightened plasticity and may determine their sensitivity to peripheral immune signals. In rodents, microglia originate from yolk sac–derived progenitors and colonize the CNS during embryogenesis, followed by a highly dynamic early postnatal period characterized by intense synaptic pruning, circuit refinement, and establishment of stable homeostatic transcriptional programs. In humans, analogous processes begin during fetal development and extend into early childhood, coinciding with periods of rapid synaptic overproduction and elimination and maturation of immune–neural interactions [152]. This developmental window represents a critical period of microglial plasticity during which environmental and immune stimuli may exert long-lasting effects on immune set points and neuroimmune programming [153]. Within this framework, the timing of BCG vaccination may be of functional relevance. Neonatal BCG administration occurs during a period in which both the immune system and microglia are still undergoing maturation and calibration. This raises the possibility that early-life immune stimulation could, in principle, imprint longer-lasting effects on innate immune programming via epigenetic reprogramming of hematopoietic and myeloid lineages and downstream modulation of peripheral–central immune communication. In contrast, most human studies assessing BCG-associated systemic or neuroimmune effects are performed in adults or elderly individuals, in whom microglia are already fully differentiated and exhibit age-related priming. In these cases, observed effects are more likely to reflect systemic immunomodulation rather than direct shaping of neurodevelopmental trajectories. Importantly, current evidence remains insufficient to determine whether neonatal versus later-life BCG vaccination leads to differential long-term CNS outcomes or altered susceptibility to neurodegenerative disease, and this remains an important open research question.

Increasing evidence indicates that microglia can exhibit both trained immunity and tolerance, depending on prior stimulation [43,154,155]. These states are induced not only by local CNS signals but also by peripheral mediators such as cytokines, hormones, metabolites, and extracellular vesicles (EVs) [113,156]. Additional modulation arises from gut–brain axis signaling, where microbiota-derived metabolites influence microglial responses to pathological proteins such as Aβ and α-synuclein [157,158]. Peripheral cytokines (e.g., IL-1β, IL-6, TNF-α) may cross or signal across the blood–brain barrier, particularly in regions with reduced integrity, thereby directly affecting CNS function [22,135]. Systemic or chronic inflammation promotes microglial priming, exaggerated responses, NLRP3 inflammasome sensitization, and a shift toward pro-inflammatory M1-like phenotypes [42,159].

Microglial activation is mediated by pattern recognition receptors (TLR2, TLR4, TLR6) recognizing PAMPs and DAMPs, but these same pathways are also triggered by endogenous neurodegenerative ligands such as Aβ, α-synuclein, mutant HTT, SOD1, and S100A8/A9 complexes [160]. A central mediator of this process is HMGB1, released primarily by microglia, which activates TLRs and RAGE receptors, driving NF-κB signaling and sustained production of IL-1β, TNF-α, and IL-6 [24]. Microglial function is further regulated by TREM2 signaling, which promotes a neuroprotective M2-like phenotype via DAP10/DAP12 pathways, enhances phagocytosis of Aβ, and suppresses inflammatory gene expression (IL-1β, IL-6, MCP-1, iNOS) [24].

Extracellular vesicles (EVs) represent another key mechanism linking peripheral immunity and CNS function, transporting RNAs and proteins capable of modulating microglial activation and propagating pathological proteins such as Aβ and α-synuclein [161,162,163,164,165]. MicroRNA-mediated activation of TLR7/8 (e.g., let-7, miR-21, miR-29a) further amplifies neuroinflammatory signaling [166]. Gut-derived EVs and metabolites (SCFAs, indoles, lactate) also influence microglial inflammatory tone via AhR and NLRP3 pathways [167,168,169,170,171,172,173,174].

Systemic inflammation, including dysbiosis-associated endotoxemia, increases circulating LPS, promoting BBB disruption and neurotoxicity, and has been linked to amyloid and tau pathology in AD and PD [175,176,177,178,179]. A key inflammatory hub in Alzheimer’s disease is the NLRP3 inflammasome, activated by Aβ species via NF-κB priming and inflammasome assembly, leading to IL-1β and IL-18 maturation [180,181,182]. Chronic activation impairs microglial phagocytosis and promotes amyloid accumulation [122,143], reflected clinically by biomarkers such as NfL, pTau231, sTREM2, and GFAP [160,181,183,184].

Microglia display functional plasticity along a continuum between pro- and anti-inflammatory states, while astrocytes (≈20% of brain cells) actively regulate CNS homeostasis, including ion balance, neurotransmission, and BBB integrity [185,186,187,188,189,190]. Upon immune stimulation, astrocytes adopt neurotoxic A1 or neuroprotective A2 phenotypes, with A1 astrocytes contributing to neuronal injury and synaptic dysfunction [191,192,193]. Importantly, peripheral immune training or tolerance can differentially affect CNS outcomes: while trained immunity may enhance pathogen clearance peripherally, in the CNS it may exacerbate neuropathology, whereas tolerance may be protective [155]. Experimental studies further demonstrate that repeated immune stimulation induces distinct epigenetic and cytokine profiles in brain versus blood, accompanied by microglial and astrocytic reprogramming via chromatin modifications (H3K4me1, H3K27ac) and metabolic switching toward glycolysis [155].

## 6. Clinical Implications and Public Health Consequences

The concept of a “neuroprotective vaccine strain” represents a potential shift in secondary prevention strategies for individuals exhibiting chronic low-grade inflammation, a state increasingly recognized as a driver of accelerated neurodegeneration and cognitive decline [194,195,196]. BCG-induced trained immunity provides a plausible biological framework for such an approach, as the vaccine generates durable epigenetic and metabolic reprogramming of innate immune cells, restoring more efficient inflammatory control while improving responsiveness to subsequent challenges [3,44]. These immune recalibrations appear particularly relevant for older adults, individuals with obesity or metabolic syndrome, and patients with chronic inflammatory disorders—groups in which immunosenescence, chronic low-grade systemic inflammation (inflammaging), and disrupted cytokine signaling contribute to vulnerability of the central nervous system [197,198]. Older cohorts may benefit disproportionately because BCG has been shown to increase cytokine responsiveness (IL-1β, IL-6, TNF-α) while simultaneously reducing baseline systemic inflammation in aging populations, thereby counteracting key features of immunosenescence [44,103,199]. Similar mechanisms could benefit individuals with obesity or metabolic syndrome, where metabolic inflammation and dysregulated innate immunity accelerate neurodegenerative pathways [200]. Furthermore, in chronic inflammatory disorders, such as autoimmune conditions, BCG has demonstrated the ability to normalize immune network functioning and promote long-term anti-inflammatory signaling [201,202], suggesting potential cross-domain benefits for neuroimmune regulation.

Safety considerations are central when imagining broader clinical implementation. Although BCG has an extensive safety record spanning over a century and billions of doses, specific risks must be recognized in the context of neuroprotective use. Immunosenescent individuals may experience altered reactogenicity or reduced immunogenicity, though current evidence shows that older adults still mount strong trained immunity responses after vaccination [203]. Autoimmunity concerns, often raised in theoretical discussions, have not been supported by empirical data. Trials in type 1 diabetes and multiple sclerosis have demonstrated favourable safety profiles without evidence of increased autoimmune activation [202,204,205]. Interactions with other vaccines, especially those using strong adjuvants, are biologically plausible due to overlapping innate immune pathways, though current data suggest synergistic rather than antagonistic effects, consistent with broader research on non-specific vaccine effects [206,207,208].

Regional differences represent another critical layer of complexity. Variability in BCG sub-strains across countries results in quantifiable differences in immunogenicity, cytokine induction, and trained immunity phenotypes due to historical genome deletions and laboratory adaptations [50,209]. Furthermore, populations in areas with high exposure to *M.tb* or routine childhood BCG vaccination may possess baseline immune priming distinct from low-endemic regions, potentially altering responses to adult revaccination [2]. Similarly, environmental mycobacteria, common in certain climates, can modulate immune reactivity and, by extension, BCG’s off-target effects [210].

Another influential factor is the microbiome, which modulates vaccine responsiveness through effects on innate immune training, cytokine signaling, and metabolic pathways. Emerging evidence shows that specific bacterial taxa predict the strength of BCG-induced cytokine responses, suggesting that microbiome variation may be a major determinant of neuroprotective efficacy [48,211]. Prior infections may also shape the immune system’s epigenetic landscape, influencing how effectively BCG can induce trained immunity [212]. These interactions highlight the need for personalized or stratified approaches to future clinical trials.

From a public health standpoint, the repurposing of BCG for neuroprotection raises strategic questions regarding allocation, target groups, and potential trade-offs. Populations displaying chronic metabolic or inflammatory stress, such as older adults, individuals with obesity, patients with chronic inflammatory illnesses, may represent high-yield candidates for immunomodulatory interventions grounded in trained immunity [206]. Integrating BCG into preventive frameworks would require robust safety monitoring, sub-strain standardization, and harmonized guidelines due to the vaccine’s variable global use patterns [213]. Nonetheless, given that non-specific effects of vaccines have previously influenced infectious disease and mortality outcomes at the population level [68,214], the broader immunological and neurological implications of BCG warrant serious consideration in public health planning.

Together, these findings underscore the need for well-designed, long-term clinical trials stratified by age, comorbidities, microbiome composition, and regional epidemiological factors. As mechanistic understanding deepens, BCG may become a model for leveraging trained immunity as a public health tool that extends beyond infectious disease prevention, potentially reshaping approaches to neurodegeneration in aging societies.

## 7. Conclusions and Future Perspectives

The accumulating evidence linking BCG vaccination to neuroprotective effects suggests a compelling convergence of innate immune training, adaptive immune recalibration, and modulation of neuroimmune communication. Among the mechanisms proposed, the induction of trained immunity appears the most robustly supported. BCG has been shown to induce long-lasting epigenetic and metabolic reprogramming in hematopoietic stem and progenitor cells, resulting in a modified innate immune set point characterized by controlled yet enhanced responsiveness to secondary stimuli [215]. Such durable alterations influence peripheral cytokine milieus and, through humoral and cellular signaling pathways, extend to neuroimmune interfaces such as the choroid plexus, meninges, and perivascular macrophage compartments. These pathways align with findings in APP/PS1 transgenic mouse models co-expressing human KM670/671L-mutated amyloid precursor protein (APP) and M146L-mutated presenilin 1 (PS1), which demonstrated that BCG vaccination improves cognitive function, recruits inflammation-resolving monocytes to neural tissue, restores regulatory T-cell balance, and increases neurotrophic factors even without significant effects on amyloid burden [10].

Adaptive immune modulation constitutes a second mechanistic pathway. BCG vaccination has been reported to adjust polarization of T-helper lymphocytes, normalize dysregulated T-regulatory cell populations, and enhance IFN-γ production in both animal and human studies [49,73]. Importantly, these effects appear to counteract features of immunosenescence, as older adults, despite diminished adaptive responsiveness, still exhibit robust induction of trained immunity cytokines (IL-1β, Il-6, TNF-α) and IFN-γ following administration [199]. Simultaneously, BCG paradoxically reduces chronic basal systemic inflammation in the elderly, with significant decreases across dozens of inflammatory proteins including chemokines, cytokines, and matrix metalloproteinases [101]. This dual action-downregulation of chronic inflammation and enhancement of stimulus-specific innate responses may represent the key biological rationale for neuroprotection in aging or metabolically dysregulated populations.

Despite compelling mechanistic evidence, the clinical landscape remains heterogeneous. Observational studies in bladder cancer patients treated with intravesical BCG have repeatedly demonstrated decreased incidence of Alzheimer’s disease, with some cohorts showing reductions of up to 30–40% [188,189,190]. These findings, though striking, are limited by a non-randomized design, specific patient populations, and distinct routes of administration. Interventional trials in older adults have confirmed immunological changes consistent with trained immunity and reduced basal inflammation, but few studies have yet linked these effects to long-term cognitive outcomes [216,217]. Trials involving autoimmune disorders, such as multiple sclerosis and type 1 diabetes, provide indirect support for durable immunometabolic reprogramming by BCG [202,218], although translating such findings to neurodegenerative settings requires caution.

Emerging Alzheimer’s and Parkinson’s-specific trials, while conceptually promising, remain in early stages. Some pilot studies have reported favorable shifts in neuroinflammatory biomarkers or slowed cognitive decline, whereas others have found minimal or inconsistent effects. Variability in host factors—metabolic status, age, microbiome composition, prior mycobacterial exposure, and genetic background—may substantially influence outcomes. For example, variation in gut microbial taxa such as *Roseburia*, *Ruminococcus*, or *Eggerthella lenta* has been shown to modulate cytokine responses to BCG, indicating that differential microbiome status could meaningfully alter neuroprotective potential [219]. Additionally, strain heterogeneity remains a significant knowledge gap. Different BCG sub-strains (e.g., Tokyo, Pasteur, Danish, Moreau) possess distinct genomic deletions that influence immunogenicity, reactogenicity, and trained immunity effects [50,209]. Determining whether certain strains provide superior neuroprotective profiles will be essential for future translational work.

Looking ahead, several priorities emerge. Randomized controlled trials must incorporate integrated immunological, neurological, and biomarker endpoints, ideally over multiyear follow-up periods capable of capturing meaningful changes in cognitive trajectories. Stratification based on inflammatory biomarkers, metabolic profiles, microbiome signatures, prior mycobacterial exposure, and genetic susceptibility will be critical to reduce noise and identify subgroups most likely to benefit. Comparative analyses of BCG sub-strains are urgently needed to clarify which strains optimize safety, anti-inflammatory effects, and trained immunity relevant to neuroprotection. Development of surrogate endpoints, including inflammatory proteomic signatures, neuroimaging markers of microglial activation, and CSF-derived immune-neutral mediators, may accelerate future trials and help elucidate mechanistic pathways.

In conclusion, BCG vaccination represents an unusual yet promising candidate for repurposing as a neuroprotective intervention. The mechanistic rationale is increasingly coherent, combining innate immune training, adaptive immune normalization, systemic anti-inflammatory shifts, and modulation of the immune–brain axis. While early observational and immunological studies provide strong biological signals, definitive clinical evidence remains limited and sometimes inconsistent. The next decade of mechanistically informed, rigorously controlled clinical research will determine whether BCG can be effectively integrated into strategies for preventing or slowing fundamental understanding of how peripheral immune modulation can influence brain aging and long-term neurological resilience.

## Figures and Tables

**Table 1:** Tuberculosis, BCG therapy, and risk of neurodegenerative diseases.

Study	Population	Exposure	Outcome	Conclusions	Limitations
Peng et al., 2015 [69]	6473 TB patients; 25,890 controls. Mean age 59.7 years; majority male.	History of TB	Pulmonary TB: 1.21-fold higher dementia risk; higher risk in men and age 50–64.	Higher dementia risk after TB.	More comorbidities; group heterogeneity; no demographic/education data; no anti-TB treatment assessment.
Shen et al., 2016 [46]	121,951 TB patients; 487,800 non-TB controls.	History of TB	1.38-fold higher Parkinson’s disease risk.	Increased Parkinson’s risk after TB.	Risk decreased over time; comorbidities; no lab/imaging data; no TB stage info.
Yeo et al., 2024 [62]	50,182 TB survivors; 50,182 controls.	History of TB	AD: 1.11-fold higher; vascular dementia: 1.48-fold higher.	Significantly higher dementia risk after TB.	No TB severity/location; no baseline cognition; no environmental/genetic data; short follow-up.
Gofrit et al., 2019 [70]	1371 bladder cancer patients; 878 received BCG.	Intravesical BCG therapy	2.4% AD in BCG vs. 8.9% without BCG.	Over 4-fold lower AD risk with BCG.	Selection bias; retrospective; ICD coding reliance; no dose–response.
Klinger et al., 2021 [71]	12,185 bladder cancer patients; 2301 received BCG.	Intravesical BCG therapy	Reduced AD risk (notably ≥75 yrs); 28% lower Parkinson’s risk.	Lower AD and Parkinson’s risk.	Patient qualification bias.
Kim et al., 2021 [72]	1290 bladder cancer patients.	Intravesical BCG therapy	60% lower AD/other dementia risk; greater reduction with induction + maintenance.	Reduced neurodegenerative disease risk.	Small sample size.
Weinberg et al., 2023 [65]	6467 bladder cancer patients; 3388 BCG; 3079 controls.	Intravesical BCG therapy	Reduced mortality and lower AD-related dementia incidence.	Reduced neurodegenerative disease risk.	No BCG dosage data.
Wang et al., 2023 [73]	38,934 bladder cancer patients; 6496 received BCG.	Intravesical BCG therapy	HR 0.88 for dementia; HR 0.89 for AD; benefit in elderly and women.	Slightly reduced neurodegeneration risk.	No randomization; lower risk also seen in non-BCG bladder cancer survivors.
Umar et al., 2024 [74]	47,947 participants.	Intravesical BCG therapy	26% reduction in AD risk; benefit in women and >75 yrs.	Lower AD risk in women and elderly.	Few studies; survival bias; limited generalizability (non-mandatory BCG countries).

Abbreviations: AD—Alzheimer’s Disease; BCG—the Bacillus Calmette–Guérin vaccine; HR—hazard ratio; ICD—International Classification of Diseases; TB—tuberculosis.

## Data Availability

The original contributions presented in this study are included in the article. Further inquiries can be directed to the corresponding author.

## Abbreviations

The following abbreviations are used in this manuscript:

AD	Alzheimer’s disease
Akt	Serine/threonine protein kinase
Aβ	Amyloid β
BBB	Blood–brain barrier
BCG	Bacillus Calmette–Guérin
BDNF	Brain-derived neurotrophic factor
CNS	Central nervous system
CT	Computed tomography
DAM	Disease-associated microglia
DAMP	Danger-associated molecular patterns
DHCR24	24-Dehydrocholesterol reductase
EV	Extracellular vesicle
GABA	Gamma-aminobutyric acid
GFAP	Glial fibrillary acidic protein
GMCSF	Granulocyte-macrophage colony-stimulating factor
HDAC	Histone deacetylase
HMGB1	High mobility group box 1
HSC	Hematopoietic stem cell
HSPC	Hematopoietic stem and progenitor cell
IFN	Interferon
IGF-1	Insulin-like growth factor
IL	Interleukin
iNOS	Inducible nitric oxide synthase
IRF8	Interferon regulatory factor 8
KDM	Histone lysine demethylase
Lnc-2	Lipocalin-2
LPS	Lipopolysaccharide
LRR	C-terminal leucine-rich repeats
MCP-1	Monocyte chemotactic protein 1
MMP	Matrix metalloproteinase
MRI	Magnetic resonance imaging
mTOR	Mechanistic target of rapamycin
NfL	plasma Neurofilament light chain
NLRP3	NLR family pyrin domain containing 3
NO	Nitric oxide
NOD	nucleotide-binding domain
NOD2	Nucleotide-binding oligomerization domain-containing protein 2
PAMP	Pathogen-associated molecular patterns
PD	Parkinson’s disease
PET	Positron emission tomography
PSP	Progressive supranuclear palsy
PRC2	Polycomb repressive complex 2
PRR	Pattern recognition receptor
RAGE	Receptor for advanced glycation end-products
ROS	Reactive oxygen species
SCFA	Short chain fatty acid
TB	Tuberculosis
TBM	Tuberculous meningitidis
TET	Ten-eleven translocation (TET) family proteins
TGF	Transforming growth factor
TLR	Toll-like receptor
TNF	Tumor necrosis factor
TREM2	Triggering receptor expressed on myeloid cells 2
α-syn	A-synuclein
